# Recombinant heat shock protein 70 in combination with radiotherapy as a source of tumor antigens to improve dendritic cell immunotherapy

**DOI:** 10.3389/fonc.2012.00149

**Published:** 2012-10-29

**Authors:** Yu-Shan Wang, Shih-Jen Liu, Su-Chen Huang, Chao-Chun Chang, Yi-Chun Huang, Weng-Lam Fong, Mau-Shin Chi, Kwan-Hwa Chi

**Affiliations:** ^1^Department of Radiation Therapy and Oncology, Shin Kong Wu Ho-Su Memorial HospitalTaipei, Taiwan; ^2^Department of Animal Science, National Ilan UniversityIlan, Taiwan; ^3^Vaccine Research and Development Center, National Health Research InstitutesMiaoli, Taiwan; ^4^School of Medicine and Institute of Radiation Science and Image Research, National Yang-Ming Medical UniversityTaipei, Taiwan

**Keywords:** dendritic cells, heat shock protein 70, radiotherapy, immunotherapy, tumor microenvironment

## Abstract

Local radiotherapy (RT) plus intratumoral dendritic cell (DC) injection can mediate immunological response. We hypothesized that co-injection of exogenous recombinant heat shock protein 70 (rHsp70) in combination with RT-DC could be as effective as co-injection of HSP-peptide for evoking specific immune response. rHsp70-prostate-specific antigen (rHSP70C′-PSA) and α-fetoprotein (rHSP70C′-AFP) were used to compare specific response. Growth inhibition of the tumor and the systemic anti-tumor immune response were measured on CT26/PSA and CT26/AFP mice model. Intratumoral co-injection of rHsp70 and DC into the irradiated tumor site induced a more potent anti-tumor immune response than injection of DC alone. rHsp70 was as effective as rHsp70C′-PSA or rHsp70C′-AFP in inducing a tumor-specific cytotoxic T lymphocyte response or tumor growth delay. These results demonstrate that co-administration with rHsp70 and RT could be a simple and effective source of tumor antigens to achieve RT-DC immunotherapy protocol and easy to apply in clinical use.

## Introduction

The tumor microenvironment (TME) is an important factor in cancer treatment (Formenti and Demaria, 2009). The effects of radiotherapy (RT) and immunotherapy are theoretically synergistic and complimentary to each other (Ferrara et al., 2009; Formenti and Demaria, 2009). Creating a favorable host anti-tumor immune microenvironment by *in situ* delivery of cytokine genes into the peritumoral site before RT results in better radio-responsiveness and systemic anticancer immunity (Wang et al., 2008). On the other hand, immunotherapy given after RT takes advantages of the release of tumor antigens from apoptotic irradiated tumor cells, which might turn local apoptosis into a systemic immune response (Friedman, 2002; Lugade et al., 2008; Teitz-Tennenbaum et al., 2008).

Heat shock proteins (HSPs) are overexpressed in various conditions of stress as stress response proteins (Zhu et al., 1996) and assist with the folding of newly translated proteins and facilitate proteolytic degradation of unstable proteins. HSPs purified from a given cancer elicit protective immunity specific to that particular cancer (Bukau and Horwich, 1998; Chen et al., 2009). Tumor-specific peptides are non-covalently bound to HSPs (Udono and Srivastava, 1994; Tamura et al., 1997). HSP-peptide complexes provoke specific protective immunity in inbred mice bearing the same specific type of tumor (Chen et al., 2004). Cancer treatments, such as chemotherapy, RT, and hyperthermia, have been shown to induce expression of intracellular HSPs (Hettinga et al., 1996; Calini et al., 2003; Yang et al., 2003; Grivicich et al., 2007), which protects tumor cells from further stress, apoptosis, and DNA damage (Hettinga et al., 1996; Calini et al., 2003). Endogenous HSP-peptide complexes must be released into the TME in order to stimulate the immune system (Baronzio et al., 2006).

Dendritic cells (DCs) that have infiltrated in the TME are responsible for antigen uptake and maturation in the draining lymph nodes, and provide the basis for generating effective anti-tumor immune responses (Banchereau and Steinman, 1998). Teitz-Tennenbaum et al. (2008) reported that radiation enhances antigen acquirement by DCs, but this radiation-enhanced loading of DCs does not change the TME, although it improves cross-priming of T cells in the draining lymph nodes. HSPs can be released passively or actively (Johnson and Fleshner, 2006). Leaderless proteins, such as Hsp70, are secreted by a non-classical, vesicle-mediated secretory pathway, a process requiring ATP (Johnson and Fleshner, 2006). Hsp70 released from tumors exists as the free form or binds to adjacent cells through an autocrine or paracrine mechanism (Mambula and Calderwood, 2006). The preparation of specific HSP-peptide complexes is tedious and, theoretically, any specific immunotherapy may be failed when antigen shifts.

Most of cancer vaccines composed tumor-specific antigens and adjuvant to active immune system (Anderson and Schneider, 2007). However, single tumor-specific antigen could not apply for every clinical patient. TME may provide several important antigens, functional immunological cells and cytokines milieu for immunotherapy if manipulated appropriately (Kaufman, 2004; Petrulio et al., 2006). Thus, using irradiated TME as source of antigens may elicit more effective immunoresponse feasible for clinical application. We hypothesized that an extracellular supply of recombinant Hsp70 (rHsp70) might be as effective as individually prepared HSP-peptide complexes in eliciting specific immunity when DC immunotherapy is combined with RT.

In this study, we demonstrated that intratumoral co-injection of rHsp70 with DCs after RT is an attractive and effective immunotherapy protocol. We also found that exogenous rHsp70 was as effective as HSP-peptide complex.

## Materials and methods

### Cell lines and mice

CT-26, a murine colon carcinoma cell line derived from a BALB/c mouse, was purchased from the Culture Collection and Research Center (Hsinchu, Taiwan) and fresh batches were thawed every year. CT-26/prostate-specific antigen (PSA) and CT-26/α-fetoprotein (AFP) were the CT-26 cell line transfected with human PSA and AFP cDNA. The PSA cDNA gene (from human prostate cancer cell line, PC-3) and AFP cDNA gene (from human hepatoma cell line, Hep 3B) were cloned into the eukaryotic expression vector PCR3.1 under the control of the CMV promoter (Invitrogen, San Diego, CA, USA). Transfected cells were selected in medium containing G418 and protein expression confirmed by Western blotting. All cell lines were maintained in Dulbecco's modified Eagle medium (DMEM) supplemented with 10% fetal bovine serum (FBS), 100 ng/ml of streptomycin, and 100 U/ml of penicillin (Invitrogen). No further authentication was used, but the expression of PSA and AFP antigens was tested during the experiments. Female BALB/c mice were obtained from the National Science Council Animal Center, Taipei, Taiwan and were used between 6 and 8 weeks of age. The studies were approved by the Institutional Animal Care and Use Committee of the Shin Kong Wu Ho-Su Memorial Hospital prior to initiation.

### Expression and purification of Hsp70, Hsp70C'-PSA, AFP, and Hsp70C'-AFP

To generate the Hsp70 expression plasmid pRSETA/*Hsp70*, the *Hsp70* gene was cloned into the *BamHI* site of the pRSETA vector with His-tag (Invitrogen). The *Hsp70C′* sequence, consisting of the terminal 735 nucleotides that code for the 28 kDa carboxyl terminal fragment of Hsp70 (amino acids 397–641), was also cloned into this site, generating the plasmid pRSET/*Hsp70C′*, which served as the backbone for the construction of the tumor antigen-*Hsp70C′* expression plasmid. The DNA fragment of PSA and AFP isolated from pCRII/PSA and pCRII/AFP was ligated into pRSET/HspC' vector to produce the Hsp70C′-PSA and Hsp70C′-AFP fusion protein. All plasmids were transfected into BL21 (DE3) PLysS or BL21 (DE3) RIL (Promega, Wisconsin, USA) *E. coli* hosts, which were cultured with shaking at 37°C, and protein expression was induced using 0.1 mM isopropyl-β-thiogalactoside. The recombinant Hsp70, Hsp70C′-PSA and Hsp70C′-AFP expressed as fusion proteins carrying a His tag were analyzed by SDS polyacrylamide gel electrophoresis and Western blotting using an anti-His monoclonal antibody (Invitrogen), and were purified on a nickel column under denaturing conditions following the manufacturer's instructions (Qiagen Inc., CA, USA). A polymyxin B agarose column (Pierce, Rockfold, IL) was used to remove endotoxin. Endotoxin levels in the purified Hsp70, Hsp70C′-PSA, and Hsp70C′-AFP were tested using the Limulus amebocyte lysate assay (Associates of Cape Cod, Inc., Cape Cod, MA) and were below the detection limit of the kit (<0.1 EU/ml).

### Generation of bone marrow-derived dendritic cells

Bone marrow-derived DCs (BM-DCs) were generated as described previously (Wang et al., 2008). Briefly, BM-DCs were isolated from BALB/c mice by culturing red blood cell-depleted BM cells in complete medium (RPMI 1640 supplemented with 10% FBS, L-glutamine, and 5 mM 2-mercaptoethanol) containing 20 ng/ml of recombinant mouse GM-CSF (Peprotech, Rocky Hill, NJ, USA) at 37°C in a humidified atmosphere with 5% CO_2_ and were fed every 3rd day with medium containing fresh GM-CSF. On day 9 of culture, non-adherent cells were harvested, washed once in complete medium, and examined for DC surface marker expression [MHC class II molecule I-A^d^/I-E^d^, CD80 (B7-1), CD86 (B7-2), CD11c, and DEC205]. The BM-DCs (5−10 × 10^5^) were stained with 50 μl of FITC-conjugated antibodies in phosphate-buffered saline (PBS) containing 1% bovine serum albumin (BSA) and 0.1% azide, which also served as the washing buffer, then were subjected to fluorescence-activated cell sorting (FACS) analysis using a FASCalibur flow cytometer (BD Bioscience, San Diego, CA, USA). Cells were also stained with the corresponding isotype-matched control IgG (BD Pharmingen, San Diego, CA, USA).

### Cytokine release assay

CT26/PSA cells were treated with 75 Gy of RT and left for 24 h at 37°C for apoptosis to occur, then were co-cultured for 24 h at 37°C with day 9 BM-DCs and 10 μg/ml of BSA, Hsp70 or Hsp70C′-PSA; 10 μg/ml of polymyxin B (Sigma) was added to one set of cultures to eliminate any effect of LPS. The culture supernatants were harvested and tumor necrosis factor α(TNF-α) and interleukin (IL)-12 measured using an enzyme-linked immunosorbent assay (ELISA) (Endogen, Rockfold, IL).

### Immunofluorescence microscopy

The cells were co-cultured as described in the previous section, then were plated onto glass slides and immediately fixed with 3.7% paraformaldehyde for 15 min; this and all subsequent steps were at room temperature. The cells were washed once with PBS and blocked with blocking buffer (PBS + 3% BSA) for 30 min, then were permeabilized by incubation with 0.1% Triton X-100 (Sigma) in PBS for 15 min. The cells were next washed once with PBS, incubated for 1 h with AlexaFluor 647-conjugated anti-MHC class II monoclonal antibody (BioLegend, San Diego, CA, USA) and Alexa Fluor 488-conjugated anti-lysosomal-associated membrane protein 1 (LAMP1) monoclonal antibody (CD107a; BioLegend), followed by five washes in PBS containing 0.05% Tween 20. The slides were then mounted in aqueous mounting solution using a coverglass (Fisher Scientific) before confocal fluorescent microscopy. Images were acquired using an Olympus FV1000 microscope (Melville, NY, USA).

### Immunohistochemistry

For immunohistochemical studies, the tumor was resected and fixed in 10% formalin for 24 h. The formalin-fixed tissues were embedded in paraffin and 5 μm paraffin sections were prepared on slides precoated with 0.1% poly-L-lysine. The paraffin sections were then deparaffinzed with xylene and rehydrated in a graded series of ethanol. T cells were identified using a monoclonal anti-CD3 antibody (clone 145−2C11, BD Pharmingen). The sections were pre-incubated with a 1:20 dilution of normal rabbit serum, then sequentially incubated for 30–60 min at 37°C with optimal dilutions of anti-CD3 antibody, biotinylated rabbit anti-mouse IgG, and horseradish peroxidase-conjugated streptavidin (Zymed, South San Francisco, CA), with PBS washes between incubations. Peroxidase activity was detected using 0.02 % diaminobenzidine tetrahydrochloride containing 0.005% hydrogen peroxide. The sections were then counterstained with hematoxylin. The immunoreaction for CD3 in lymphocytes was evaluated independently by two pathologists and graded as follows: (−) = no positive cells, (+) = 1−25% of the cells stained, (++) = 26−50% of the cells stained and (+++) = 51−100% of the cells stained.

### Animal study

BALB/c mice were injected subcutaneously in the left flank with 1 × 10^5^ CT-26/PSA or CT-26/AFP tumor cells on day 0. At day 14 after injection, the mice received local irradiation with 8 Gy, then, on the following day, 5 × 10^5^ syngeneic DCs or PBS in 25 μl were injected into the tumor area. In some groups, the DCs were mixed with 50 μg of rHsp70, rHsp70C′-PSA, rAFP or rHsp70C′-AFP fusion protein before injection. The size of the tumor was measured at least three times each week and recorded as length (L) and width (W) and the tumor volumes calculated as L × W^2^/2. The tumor volume ratio was determined as the tumor volume after treatment divided by the initial tumor volume (day 14).

### Cytotoxicity T lymphocyte (CTL) assay

On day 35 after tumor injection, the mice were killed and their spleens harvested. Erythrocyte-depleted splenocytes (1 × 10^6^ cells/ml) were cultured *in vitro* with mitomycin C-treated CT26/PSA tumor cells (1 × 10^6^ cells/ml) in 24-well plates for 5 days, with 50 U/ml of recombinant human IL-2 (Proleukin; Novartis Pharmaceuticals, East Hanover, NJ) being added each day. On day 5, the cells were collected, dead cells removed on a density gradient, and the viable cells tested for specific cytotoxicity in a standard 5-hr chromium-51 (^51^Cr)-release assay. The percentage specific cytotoxicity was calculated as 100 × [(experimental release – spontaneous release)/(maximal release – spontaneous release)].

### Statistical analysis

All results were compared using an unpaired *t*-test (two-tailed) or One-Way ANOVA. Differences were considered statistically significant at a *P*-value <0.05.

## Results

### Maturation of BM-DCs is induced by rHsp70 or rHsp70C′-PSA

**Figure 1:**
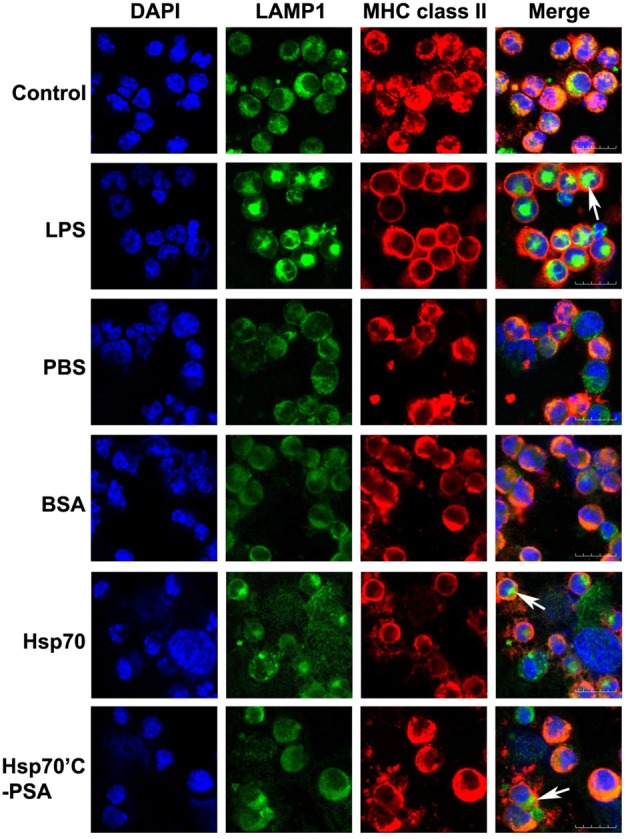
**Irradiated-CT26/PSA cells cause BM-DC maturation in the presence of rHSPs.** BM-DCs (1.5 × 10^6^ in 1 ml) were incubated with medium alone (control) or LPS (10 μg/ml) or were cocultured with irradiated-CT26/PSA cells (1.5 × 10^6^ in 1 ml) in PBS or 10 μg/ml of BSA, rHsp70, or rHsp70C′-PSA. After 48 h, the cells were harvested and stained with antibodies against MHC class II molecule I-A^d^ and LAMP1. Column 1, DAPI nuclear staining; column 2, LAMP1 staining; column 3, MHC class II intracellular staining; column 4, merged images. Images were acquired using confocal microscopy at a magnification of ×400. This experiment was repeated three times with reproducible results. Scale bars, 20 μm.

**Figure A1:**
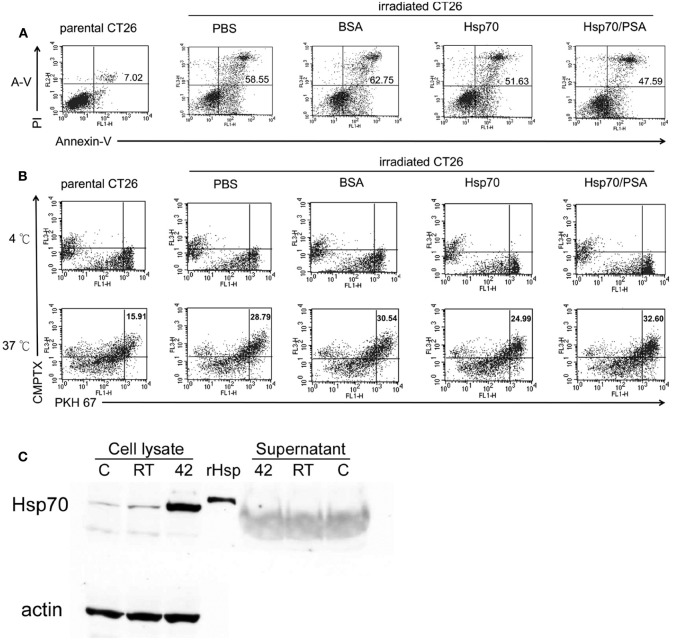
**Exogenous rHsp70 did not increase radiation–induced CT26/PSA apoptosis nor increase BM-DC phagocytosis. (A)** The percentage of apoptotic CT26/PSA cells was analyzed by annexin-V and PI (propidium iodide) staining (shown in the upper right corner) in, left to right, parental CT26/PSA cells in PBS (parental CT26) or irradiated CT26/PSA cells (irradiated CT26) in PBS (PBS) or 10 μg/ml of BSA (BSA), rHsp70 (HSP70), or rHsp70C′-PSA (Hsp70/PSA) for 24 h incubation. The number of percentage was indicated AnnexinV-positive cells. **(B)** Cell Tracker-labeled CT26/PSA (5 × 10^5^), pretreated as in Figure 1A, and PKH-67-labeled BM-DCs (5 × 10^5^) were cocultured in 6-well plates for 24 h at 4°C (top row, no phagocytosis control) or 37°C (bottom row) and the percentages of double-positive cells measured. The panels are arranged in the same order as in Figure 1A. **(C)** Untreated (lane C) or irradiated CT26/PSA cells (lane RT) or control cells heated at 42°C for 30 min (positive control; lane 42) were harvested for Western blot analysis for Hsp70 protein in the cell lysate and culture supernatant. Lane rHsp is rHsp70. These data are representative of those obtained in three experiments.

Maturation of DCs was determined by the cellular distribution of LAMP1 and MHC class II molecules and the expression of specific cell surface markers. Figure 1 shows LAMP1 and MHC class II molecule staining of immature BM-DCs that were left untreated (control row) or were co-cultured with irradiated-CT26/PSA cells in the absence (PBS and BSA rows) or presence rHsp70 or rHsp70C′-PSA. Irradiated-CT26/PSA cells alone did not induce DC maturation (surface expression of co-stimulatory molecules and MHC class II, as shown by the MHC class II antigen remaining in the cytoplasm (predominantly in lysosomes) and colocalizing with LAMP1. In contrast, in lipopolysaccharide (LPS)-treated immature BM-DCs (positive control; 10 μg/ml of LPS for 24 h) and in BM-DCs cocultured with irradiated CT26/PSA cells and rHsp70 or rHsp70C′-PSA, high levels of MHC class II antigen moved to the plasma membrane and LAMP1 was seen as clusters in the cytoplasm (white arrow), indicating maturation of BM-DCs (Chow et al., 2002; Blander and Medzhitov, 2006). We also tested for phenotypic changes on DC maturation. Table 1 shows that most of the cells were DCs (positive for CD11c and MHC class II antigen) and that rHsp70 and rHsp70C′-PSA upregulated the expression of the mouse DC maturation markers CD86 and DEC205 on BM-DCs incubated with irradiated CT26/PSA cells to a similar level. Radiation-induced apoptosis of CT-26/PSA cells was confirmed. As shown in Figure A1A. Addition of exogenous Hsp70 or Hsp70/PSA (fourth and fifth panels) did not increase the percentage of apoptotic cells after RT compared to PBS or BSA. There was no significant difference in antigen uptake rate in the presence of exogenous Hsp70 or Hsp70/PSA compared to PBS or BSA (Figure A1B). RT alone resulted in expression of low levels of Hsp70 on the CT26/PSA cells which was not released into the culture supernatant (Figure A1C).

**Table 1:** **DC surface marker expression after incubation with irradiated CT26/PSA cells in the presence or absence of Hsp70 or Hsp70C′-PSA^*a*^**.

**CT26/PSA (RT)**						
**Marker**	**Control**	**LPS**	**PBS**	**BSA**	**Hsp70**	**Hsp70C′-PSA**
CD11c	94 ± 3	91 ± 2	93 ± 3	95 ± 1	92 ± 4	96 ± 2
CD86	9 ± 5	35 ± 4	10 ± 3	11 ± 4	42 ± 12	67 ± 10
MHC class II	96 ± 3	93 ± 3	95 ± 2	97.2	97.8	97.5
DEC205	4 ± 4	14 ± 5	4 ± 4	4 ± 3	17 ± 10	16 ± 5

a BM-DCs (day 8 cultures) were cultured with medium (negative control) or 10 mg/ml of LPS (positive control) for 24 h or with irradiated CT26/PSA cells in PBS alone or PBS containing 10 mg/ml of BSA, Hsp70, or HspP70C'-PSA for 24 h; then the cells were harvested and stained with antibodies against MHC class II antigen (I-Ad), CD11C, CD86, or DC205 and analyzed by flow cytometry. Values are given as the percentage of gated regions after subtraction of the percentage of cells stained with normal mouse IgG. The data are the mean for three independent experiments.

### rHsp70 or rHsp70C′-PSA activates BM-DCs to release cytokines

**Figure 2:**
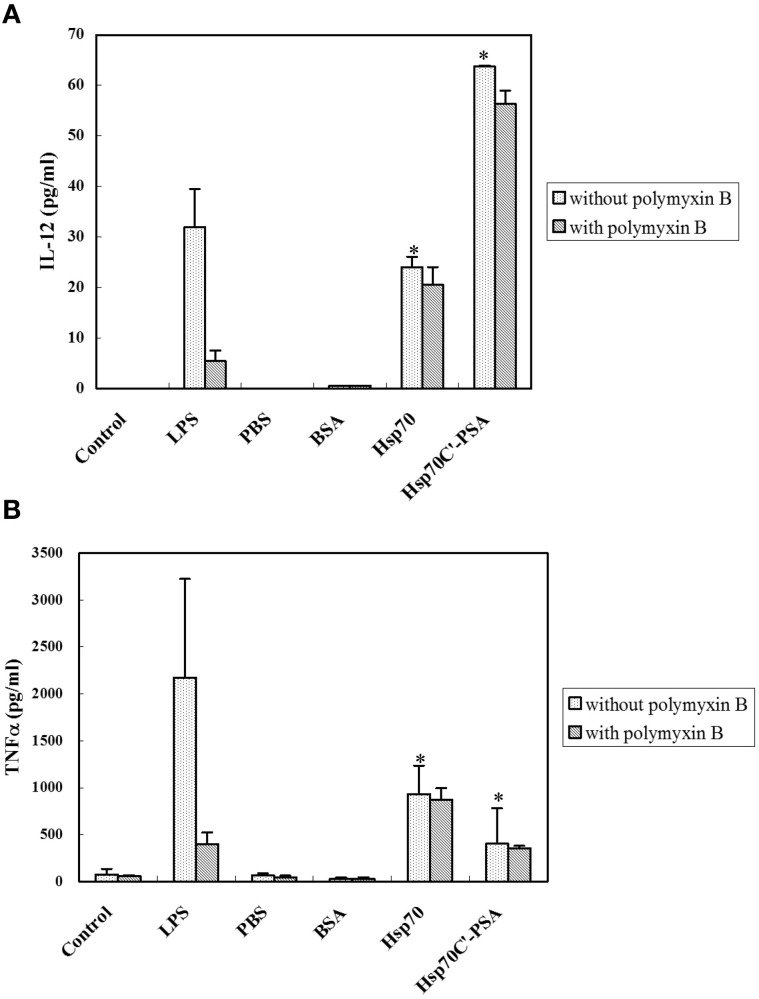
**Exogenous rHSPs enhance the expression of pro-inflammatory cytokines.** BM-DCs (1.5 × 10^6^ in 1 ml) were incubated with medium alone (control) or LPS (10 μg/ml) or were cocultured at 37°C for 24 h with irradiated-CT26/PSA cells (1.5 × 10^6^ in 1 ml) of PBS or 10 μg/ml of BSA, rHsp70, or rHsp70C′-PSA in the absence or presence of 10 μg/ml of polymyxin B. The supernatants were then harvested and the concentration of IL-12 **(A)** or TNF-α **(B)** measured by ELISA. The data are the mean ± SD for three-independent experiments. ^*^Indicates *p* < 0.05.

DC activation is characterized by cytokine release. As shown in Figure 2, incubation with irradiated CT26/PSA cells alone (PBS or BSA on Figure) did not induce IL-12 and TNF-α secretion by BM-DCs. However, addition of rHsp70 or rHsp70C′-PSA to the culture medium-induced a significant increase in IL-12 and TNF-α release, as did incubation of BM-DCs with LPS. The cytokine-releasing effects of rHsp70 and rHsp70C′-PSA were not due to endotoxin contamination, as addition of the endotoxin antagonist polymyxin B did not affect the cytokine release.

### rHsp70 or rHsp70C′-PSA enhances RT-DC immunotherapy equally

**Figure 3:**
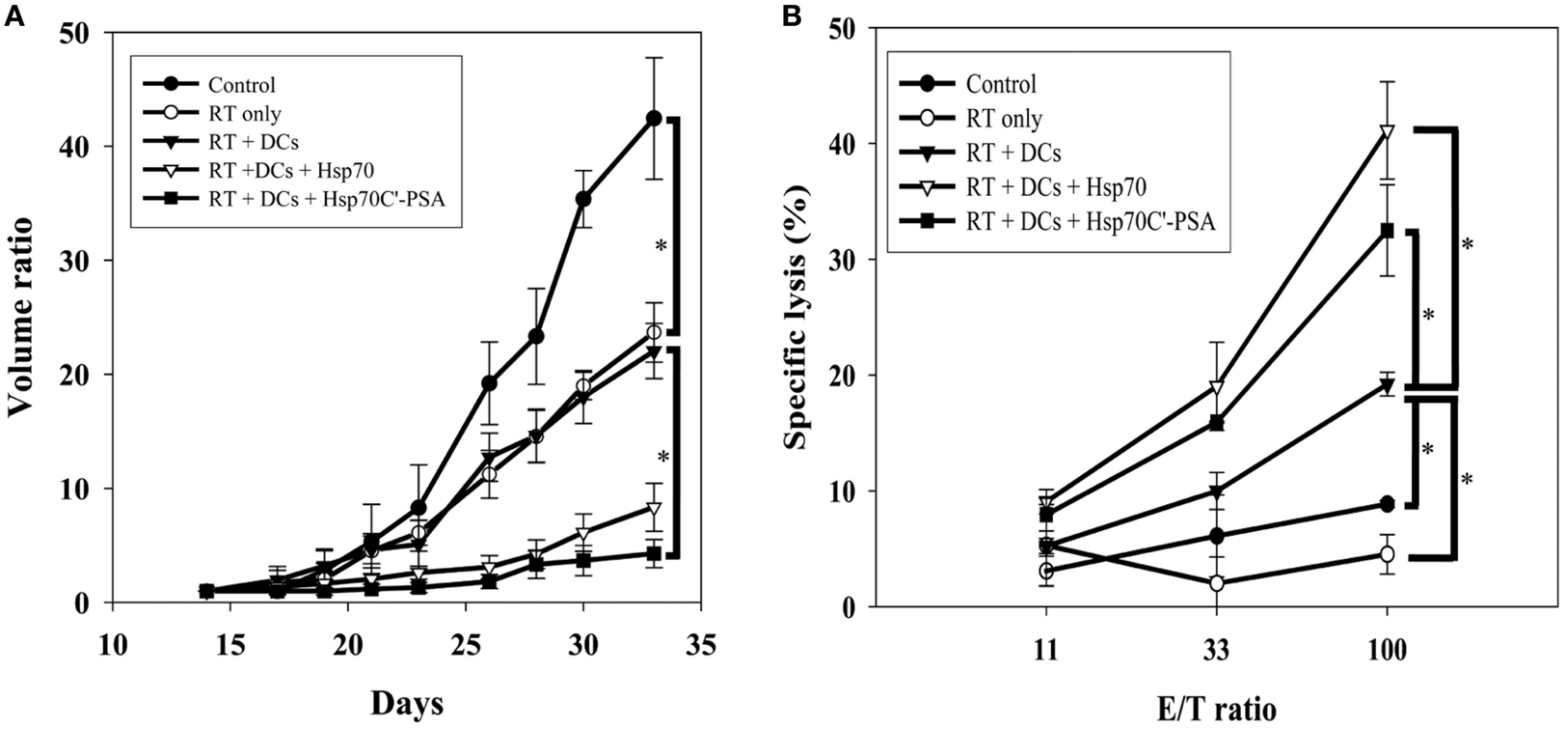
**Tumor growth inhibition, tumor-specific CTLs and tumor CD3^+^ T cell infiltration assay. (A)** Mice were injected with 1 × 10^5^ CT26/PSA tumor cells s.c. on day 0 and treated with 8 Gy of RT on day 14 followed by immunotherapy in different groups. Each treatment group included 6 mice. Mean volume ratio, with SD for each group, is plotted vs. days after tumor-cell inoculation. **(B)** Seven days after treatment immunotherapy, splenocytes were harvested for CTLs assay. The cytotoxic activity of the splenocytes were determined by ^51^Cr-release assay at various effector/target cells (E/T) ratios. The data are shown as the mean specific lysis ± SD for three experiments. ^*^Indicates *p* < 0.05.

**Figure A2:**
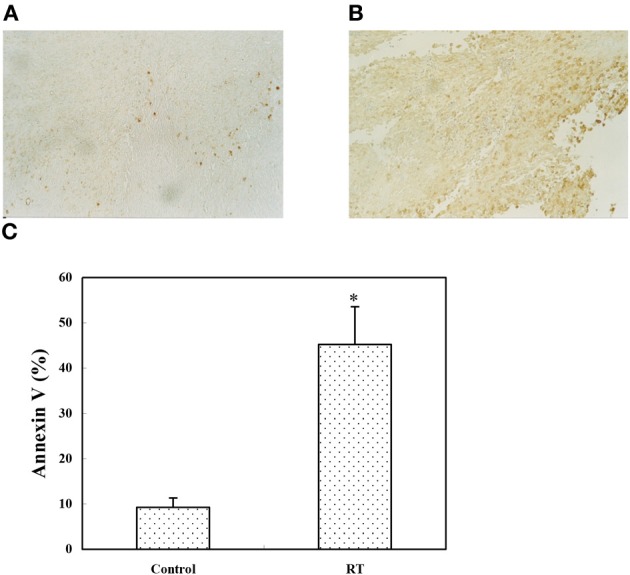
***In vivo* tumor apoptosis assay.** CT26/PSA cells (1 × 10^5^) were injected s.c. on day 0. On day 14, the tumor size was around 4 mm in diameter and the mice were left untreated **(A)** or received 8 Gy of RT **(B)**, then, on day 16, sections from the tumor site were analyzed using the TUNEL assay. Apoptotic cells are stained brown (magnification, 100 ×). **(C)** The tumor mass was digested with collagenase and analyzed by annexin V staining; the apoptotic cells are expressed as the percentage of annexin V-positive cells. The data represent the mean ± SD for three mice. ^*^Indicates *p* < 0.05.

**Figure A3:**
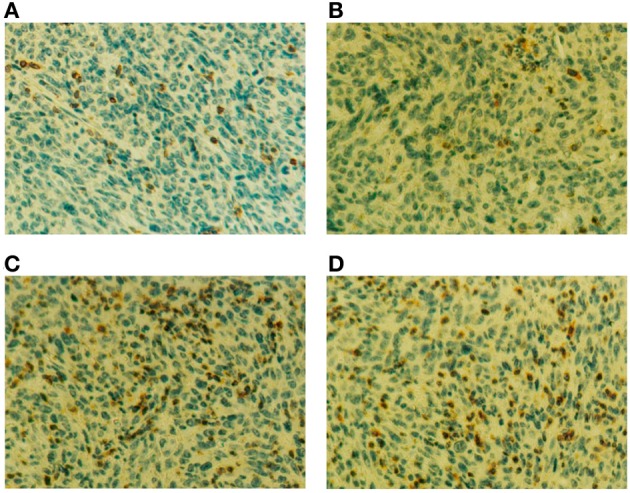
**(A–D)** Four days after RT-DC immunotherapy, the tumor were removed and analyzed by immunohistochemistry. CD3^+^ T cell infiltration is seen as brown spots in the section counterstained with hematoxylin (magnification, 400 ×). Sections are from mice treated with PBS **(A)**, BM-DCs alone **(B)** or BM-DCs plus 50 μg of rHsp70 **(C)** or rHsp70C′-PSA **(D)**.

BM-DCs alone or together with Hsp70 or Hsp70C′-PSA were injected into the tumor site 2 days after 8 Gy of irradiation. Two days later, some of the tumor mass was removed for immunohistochemical staining, and the rest mice were measured for tumor size every 2 or 3 days until death (tumor size > 3000 mm^3^). The degree of tumor apoptosis before treatment was assessed by TUNEL and Annexin V staining (Figure A2). As shown in Figure 3A, RT-DC immunotherapy delayed tumor growth, but the results did not differ from those for RT alone. However, injection of rHSPs with RT-DC immunotherapy resulted in significant growth delay compared to RT or RT-DC alone (*p* < 0.05), with no difference between groups of Hsp70C′-PSA and Hsp70. Seven days after BM-DC injection, the mice were sacrificed and their spleen cells assessed for their ability to mediate specific killing of CT26/PSA cells. As shown in Figure 3B, specific cytotoxicity was higher in mice co-injected with DCs and rHSPs than with DCs alone or RT alone, with no significant difference between the groups treated with rHsp70 or rHsp70C′-PSA. The lytic activity of the cultured spleen cells correlated well with the degree of tumor growth inhibition in each group shown in Figure 3A. RT-DC immunotherapy elicited a significant increase in T cell-mediated cytotoxicity compared to control mice, while RT alone had no significant effect. A greater infiltration of CD3^+^ T cells were observed when rHsp70 (+++) or rHsp70C′-PSA (+++) were co-injected with BM-DCs immunotherapy compared to mice injected with PBS (+) or BM-DCs (+) alone (Figure A3). There was no difference in the percentage of CD4^+^ and CD8^+^ cells in the infiltrated T cells between all groups (data not show).

### rHsp70 or rHsp70C′-AFP enhances RT-DC immunotherapy equally

**Figure 4:**
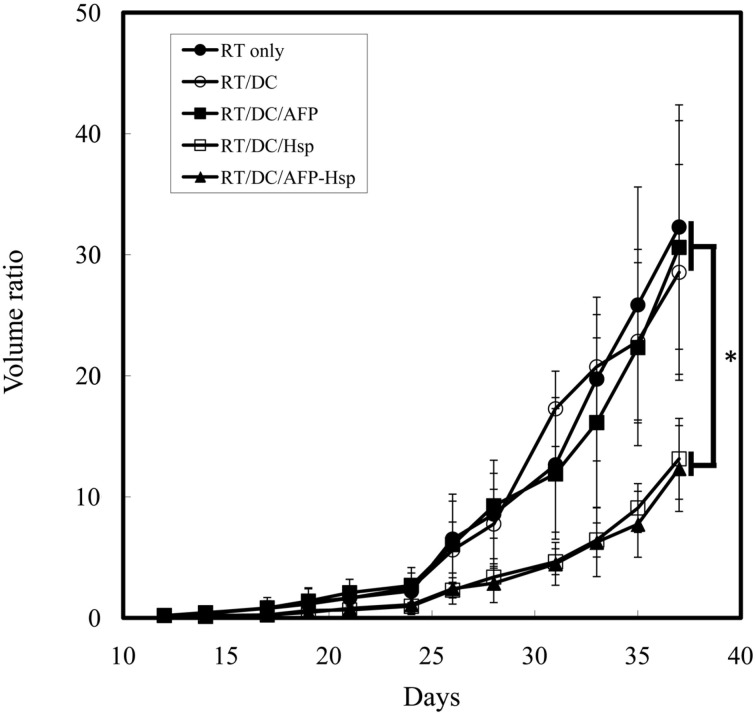
**Tumor growth inhibition assay in rHsp70/rAFP model.** Mice were injected with 1 × 10^5^ CT26/AFP tumor cells s.c. on day 0 and treated with 8 Gy of RT on day 14 followed by immunotherapy in different groups. Each treatment group included 6 mice. Mean volume ratio, with SD for each group, is plotted vs. days after tumor-cell inoculation. ^*^Indicates *p* < 0.05.

To prove rHsp70 can serve as all-purpose adjuvant instead of specific Hsp-fusion peptide, we constructed another recombinant tumor antigen (rAFP) and Hsp70 fusion protein (rHsp70C′-AFP). As shown in Figure 4, co-injection of rHsp70 or rHsp70C′-AFP with RT-DC immunotherapy significantly enhanced the growth inhibition effect than RT alone, RT-DC alone or RT-DC plus rAFP. Again, there is no difference between groups of Hsp70C′-AFP and Hsp70. The addition of rAFP did not show tumor growth inhibition indicated that the effect is not the result of a contamination in protein purification.

### rHsp70 or rHsp70C′-PSA could not enhance RT without DC

**Figure 5:**
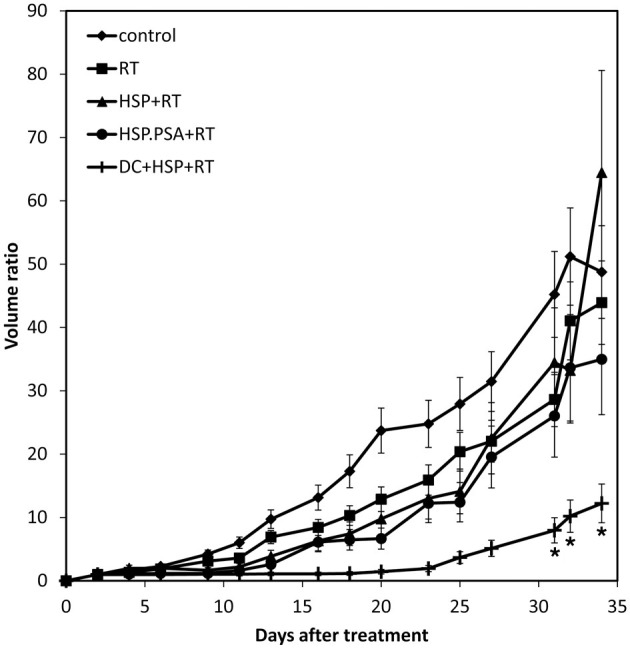
**Tumor growth inhibition assay in rHsp70/rPSA without DC.** Mice were injected with 1 × 10^5^ CT26/PSA tumor cells s.c. on day 0 and treated with 8 Gy of RT on day 14 followed by intratumoral injection 50 μg of rHsp70, or rHsp70C′-PSA. Each treatment group included 6 mice. Tumor volume (mm^3^), with SD for each group, is plotted vs. days after tumor-cell inoculation. ^*^Indicates *p* < 0.05.

In final step, we further investigated whether rHsp70 or rHsp70C′-PSA enhance RT without DC immunotherapy or not. There were no significantly differences in tumor growth inhibition between RT alone, co-injection of rHsp70 or rHsp70C′-PSA with RT (Figure 5). This result supported our hypothesis that DC plays the major role in RT enhancement and rHsp70 was served as adjuvant.

## Discussion

We have previously reported that intratumoral injection of immature DCs into the irradiated tumor (RT-DC treatment) elicits tumor-specific immunity in hepatocellular carcinoma patients (Chi et al., 2005). The present study suggests that co-injection of rHsp70 with DCs can enhance the efficacy of RT-DC immunotherapy, providing the basis for a future clinical protocol.

Irradiation of a tumor may not result in as immunogenic apoptotic cells as ultraviolet irradiation or hyperthermia (Sierra-Rivera et al., 1993; Matsumoto et al., 1995; Mukhopadhaya et al., 2007). In our study, incubation of stressed, but not non-stressed, apoptotic tumor cells with syngeneic DCs-induced protective immunity, indicating that uptake of apoptotic cells by DCs alone may not efficient enough to activate the immune response, as reported by others (Feng et al., 2002). The presence of Hsp70 on the surface of heat-stressed tumor cells plays a critical role in enhancing their immunogenicity (Feng et al., 2002). The secondary signals needed to activate DC function are induced by danger molecular pattern proteins, such as calreticulin, HSPs, and high mobility group box 1 (Brusa et al., 2009). HSPs are generally regarded as protectors of cells against various stresses, such as ischemia, heat, chemotherapy, or radiation (Ciocca and Calderwood, 2005). However, HSPs are also associated with the processing of antigenic peptides and their translocation from the cytosol to the endoplasmic reticulum (Hartl and Hayer-Hartl, 2002). HSP-peptides or HSP-antigen genes have been successfully used as vaccine adjuvants in adaptive immunity (Chen et al., 2000; Zeng et al., 2006; Zhang et al., 2007). However, there have not been any reports of the use of exogenous recombinant HSP, together with radiation, to modulate the local tumor response and immune response.

The exogenous addition of rHsp70 or rHsp70 fusion proteins did not enhance the phagocytic ability of DCs *in vitro*, but resulted in upregulation of co-stimulatory molecules on the DC surface with DC maturation. Secretion of IL-12 and TNF-α was induced by adding rHsp70 or rHsp70C′-PSA to the irradiated tumor cell/DC cultures. These data are consistent with those from other studies (Gallucci et al., 1999; Lutz et al., 1999; Feng et al., 2002) showing that apoptotic cells cannot stimulate DC activation efficiently in the absence of inflammatory signals. Candido et al. (2001) reported that intratumoral administration of BM-DCs can partially inhibit the growth of an established tumor, but co-administration of TNF-α leads to a greater DC-mediated anti-tumor effect.

The finding that co-injection of DCs and rHsp70C′-PSA or rHsp70C′-AFP did not result in a greater CTL response than co-injection of DCs and rHsp70, both *in vitro* and *in vivo*, may be important, as it suggests that recombinant HSP can serve as an all-purpose adjuvant in RT-DC immunotherapy. It seems that the anti-tumor immunity provoked by RT-DC is not PSA or AFP-specific, but CT-26 cell-specific. In a parallel CTL assay using CT-26 parental cells as targets, addition of rHsp70 or rHsp70C′-PSA resulted in comparable lysis to that seen using CT-26/PSA cells as targets (data not shown). Unidentified tumor-specific antigens more immunogenic than PSA or AFP may also exist. As antigen shift is always a problem in immunotherapy, the therapeutic effect of specific immunotherapies may only last for a while. The combination of RT, as both a tumorcidal method and as a releaser of tumor antigens, with an all-purpose adjuvant, such as HSPs and DCs, may have been clinically useful. RT can be easily delivered to tumor sites more precisely than most other clinical modalities. Site-directed injection of immature DCs into apoptotic tumor cells a few days after RT has proved clinically feasible and effective. Exogenous addition of recombinant HSPs can further activate signals to prime the CTL response. We have reported, hepatoma may be a suitable target for this protocol. We are now applying for a clinical trial on high-risk prostate cancer which can be suitable for hypofractionated RT.

In summary, we have found an efficient method for turning radiation-induced local apoptosis into a systemic anti-tumor immune response. Co-injection of rHsp70 and DCs into the irradiated tumor site induced a more potent anti-tumor immune response than injection of DCs alone. rHsp70 was as effective as rHsp70C′-PSA or rHsp70C′-AFP in inducing a tumor-specific CTL response or tumor growth delay. The combined use of RT-DC and recombinant HSP as an all purpose adjuvant may convert non- or low-immunogenic tumor cells to higher immunogenicity through promotion of DC function.

### Conflict of interest statement

The authors declare that the research was conducted in the absence of any commercial or financial relationships that could be construed as a potential conflict of interest.

## Appendix

### Apoptosis of irradiated CT-26/PSA cells and phagocytosis

CT-26/PSA cells were stained with PKH 67 (Sigma, St Louis, MO, USA) according to the manufacturer's protocol, then the labeled tumor cells were irradiated to induce apoptosis. Apoptosis was confirmed at 24 h after RT using an Annexin V Apoptosis Kit (BD Pharmingen). Day 9 BM-DCs stained with PKH-67 (5 × 10^5^ in 1 ml) were cocultured with γirradiated CT-26/PSA cells stained with Cell Tracker (Molecular Probes, Eugene, OR, USA) (5 × 10^5^ in 1 ml) for 24 h at either 4°C (no phagocytosis control) or 37°C (experimental group), then phagocytosis by BM-DCs was analyzed by flow cytometry.

### Western blot analysis

For protein analysis, cells were lysed for 5 min at room temperature in a buffer composed of 150 mmol/L NaCl, 50 mmol/L Tris (pH 8.0), 5 mmol/L EDTA, 1% (v/v) Nonidet P-40, 1 mmol/L of phenylmethylsulfonyl fluoride, 20 μg/ml of aprotinin, and 25 μg/ml of leupeptin and the protein concentration of the lysates measured using Bio-Rad protein assay reagent. Cell lysates (100 μg) were electrophoresed on a 12% SDS polyacrylamide gel and the proteins transferred to an Immobilon-P PVDF membrane (Millipore, Bedford, MA), which was then blocked for 2 h at room temperature in PBS-0.05% Tween 20 containing 10% nonfat milk, then incubated with anti-Hsp70 antibodies (SPA810; Stressgen, San Diago, CA) for 2 h at room temperature in PBS-0.05% Tween 20 containing 5% nonfat milk, then for 1 h at room temperature with horseradish peroxidase-conjugated secondary antibodies (Jackson ImmunoResearch Laboratories, West Grove, PA) in the same buffer. Blots were developed using a chemiluminescent detection system (ECL; GE Life Science, Buckinghamshire, UK).

#### Apoptosis assessment

Apoptosis in the tumor tissue was assessed using the terminal deoxynucleotidyl transferase-mediated dUTP-biotin nick end-labeling (TUNEL) assay performed using a commercial TdT-FragEL™ DNA fragmentation detection kit (Oncogene Research, Darmstadt, Germany). Briefly, tumor tissue was excised from the mouse and embedded in paraffin sections. After hydration and dehydration, the slides were fixed with 4% paraformaldehyde in PBS overnight and permeabilized with 20 mg/ml of proteinase K in 10 mM Tris pH 8 for 10 min at room temperature. Endogenous peroxidases were inactivated by incubation with 3% H_2_O_2_ in methanol for 5 min at room temperature, then, after washing, the cells were incubated with biotin-labeled dNTPs in the presence of terminal deoxynucleotidyl transferase enzyme solution for 1.5 h at 37°C and biotinylated nucleotides detected by incubation with a streptavidin-horseradish peroxidase conjugate for 25 min at room temperature, followed by incubation with diaminobenzidine for 15 min at room temperature. Finally, tumor cells were counterstained with methyl green for 3 min at room temperature.
